# Improved Survival and Initiation of Differentiation of Human Induced Pluripotent Stem Cells to Hepatocyte-Like Cells upon Culture in William’s E Medium followed by Hepatocyte Differentiation Inducer Treatment

**DOI:** 10.1371/journal.pone.0153435

**Published:** 2016-04-13

**Authors:** Minoru Tomizawa, Fuminobu Shinozaki, Yasufumi Motoyoshi, Takao Sugiyama, Shigenori Yamamoto, Naoki Ishige

**Affiliations:** 1 Department of Gastroenterology, National Hospital Organization, Shimoshizu Hospital, 934–5 Shikawatashi, Yotsukaido City, Chiba 284–0003, Japan; 2 Department of Radiology, National Hospital Organization, Shimoshizu Hospital, 934–5 Shikawatashi, Yotsukaido City, Chiba 284–0003, Japan; 3 Department of Neurology, National Hospital Organization, Shimoshizu Hospital, 934–5 Shikawatashi, Yotsukaido City, Chiba 284–0003, Japan; 4 Department of Rheumatology, National Hospital Organization, Shimoshizu Hospital, 934–5 Shikawatashi, Yotsukaido City, Chiba 284–0003, Japan; 5 Department of Pediatrics, National Hospital Organization, Shimoshizu Hospital, 934–5 Shikawatashi, Yotsukaido City, Chiba 284–0003, Japan; 6 Department of Neurosurgery, National Hospital Organization, Shimoshizu Hospital, 934–5 Shikawatashi, Yotsukaido City, Chiba 284–0003, Japan; University of Tampere, FINLAND

## Abstract

**Background:**

Hepatocyte differentiation inducer (HDI) lacks both glucose and arginine, but is supplemented with galactose and ornithine, and is added together with other reagents such as apoptosis inhibitor and oncostatin M. Although human induced pluripotent stem (iPS) cells initiate hepatocyte differentiation, most die within 7 days. In this study, we investigated both HDI and conventional media for their potential to improve cell survival.

**Materials and Methods:**

201B7 iPS cells were cultured in conventional media. This consisted of three cycles of 5-day culture in William’s E (WE) medium, followed by a 2-day culture in HDI.

**Results:**

Expression levels of α-feto protein (AFP) were higher in cells cultured in WE and in Dulbecco’s Modified Eagle’s Medium/Nutrient F-12 Ham (DF12). 201B7 cells expressed the highest AFP and albumin (ALB) when cultured in HDI for 2 days following 7-day culture in WE. After three cycles of 5-day culture in WE followed by 2 days in HDI, 201B7 cells expressed AFP and ALB 54 ± 2.3 (average ± standard deviation) and 73 ± 15.1 times higher, respectively, than those cultured in ReproFF (feeder-free condition).

**Conclusion:**

201B7 cells survived culture in WE for 7 days followed HDI for 2 days. After three cycles of culture under these conditions, hepatocyte differentiation was enhanced, as evidenced by increased AFP and ALB expression.

## Introduction

Introduction of reprogramming factors has enabled production of human induced pluripotent stem (iPS) cells [1]. iPS cells hold promise for regenerative medicine applications because these cells can potentially differentiate into somatic cells [2]. Thus, hepatocytes generated from iPS cells can be applied to treating liver insufficiencies [3].

Current protocols of hepatocyte differentiation from iPS cells rely on either sequential stimulation with growth factors or introduction of transcription factors [4–9]. The most common procedures include stepwise stimulation of iPS cells with growth factors to simulate fetal liver development [4–7]. During liver development, transcription factors upregulate the expression of genes necessary for hepatocyte differentiation [8]. iPS cells, human umbilical vascular endothelial cells, and human mesenchymal stem cells are mixed to form a liver organoid [10]. Under the influence of these transcription factors, iPS cells differentiate into hepatocytes [7, 9]. However, these protocols have few limitations, including the fact that the hepatocytes produced are immature, known as “hepatocyte-” or “hepatoblast-like” cells [11].

Glucose is an important source of energy for survival, while arginine is considered a non-essential amino acid since it is produced de novo. Cells require additional arginine owing to insufficient production [12], and cannot survive without both glucose and arginine [13]. Hepatocytes produce glucose from galactose and arginine from ornithine using galactokinase and through the urea cycle, respectively [14–16]. Meanwhile, the hepatocyte selection medium (HSM) does not contain either glucose or arginine, but is supplemented with galactose and ornithine [17]. iPS cells typically die within 3 days, but hepatocytes survive when cultured in HSM [18]. Further, hepatocyte differentiation inducer (HDI) consists of HSM supplemented with additional reagents. HDI was found to initiate the differentiation of iPS cells into hepatocytes, as demonstrated by increased expression of α-feto protein (AFP) [19]. However, most of these cells differentiating to hepatocytes die within 7 days, and not enough cells can be obtained [20].

In this study, we investigated the sequential culture of iPS cells in HDI and conventional media to optimize cell survival and yield over culture in HDI alone.

## Materials and Methods

### Cell culture

201B7 cells, a human iPS cell line, were purchased from the RIKEN Cell Bank (Tsukuba, Japan), and cultured under feeder-free conditions in Repro FF medium (Reprocell, Yokohama, Japan) on 6-well plates (Asahi Techno Glass, Funabashi, Japan) coated with Matrigel^TM^ (Becton Dickinson, Franklin Lakes, NJ, USA). The cells were incubated at 5% carbon dioxide and 37°C in a humidified chamber. They were then harvested with Accutase^®^ (Innovative Cell Technologies, Inc., San Diego, CA, USA), seeded onto fresh 6-well plates, and observed by microscopy (CKX41N-31PHP; Olympus, Tokyo, Japan). The undifferentiated 201B7 cells were passaged every 4–5 days.

### Culture in conventional media

201B7 cells were cultured in conventional media supplemented with 1.2 mg/mL nicotinamide, 30 ng/mL proline, and 10% knockout serum replacement (KSR; Life Technologies, Grand Island, NY, USA). Nicotinamide and proline were added because they are necessary for primary hepatocyte proliferation, given our initial goal to help the cells proliferate upon culture in HDI [21, 22]. The conventional media tested are listed in Table 1. The cells were cultured for 7 days.

**Table 1:** Conventional media used in this study.

Abbreviation	Name	Company	Catalog number
L15	Leibovitz's-15	Life Technologies, Grand Island, NY, USA	11415–064
DMEM	Dulbecco's Modified Eagle's Medium	Sigma-Aldrich, St. Louis, MO, USA	D5796
RPMI	Roswell Park Memorial Institute 1640	Sigma-Aldrich	R8758
WE	William’s E	Life Technologies	12551–032
DF12	Dulbecco's Modified Eagle's Medium/Nutrient F-12 Ham	Sigma-Aldrich	D6421
MEM	Minimum Essential Medium	Life Technologies	11090–081
GMEM	Glasgow’s Minimum Essential Medium	Life Technologies	11710–035
Improved MEM	Improved Minimum Essential Medium	Life Technologies	10373–017
IMDM	Iscove’s Modified Dulbecco’s Medium	Life Technologies	12440–053
CMRL	Connaught Medical Research Laboratories 1066	Life Technologies	16600–082
BME	Basal Medium Eagle	Life Technologies	21010–046
McCoy	McCoy’s 5A	Life Technologies	21010–046
MCDB	MCDB 131	Life Technologies	10372–019

### Reagents

Non-essential amino acids (glycine, 7.5 mg/L; L-alanine, 8.9 mg/L; L-asparagine, 13.2 mg/L; L-aspartic acid, 13.3 mg/L; L-glutamic acid, 14.7 mg/L; L-proline, 11.5 mg/L; and L-serine, 10.5 mg/L) and sodium pyruvate (1 mM) were purchased from Life Technologies. The apoptosis inhibitor, M5054 [100 μg/mL; 2,2′-methylenebis(1,3-cyclohexanedione)], was purchased from Merck (Billerica, MA, USA). 2-(N-(5-chloro-2-methylphenyl)methylsulfonamido)-N-(2,6-difluorophenyl)acetamide), (10 nM; hepatocyte functional proliferation enhancer, FPH1) was purchased from XcessBio (San Diego, CA, USA) [23]. Galactose (900 mg/mL), ornithine (1 mM), oncostatin M (20 ng/mL), nicotinamide (1.2 mg/mL), proline (30 ng/mL), and L-glutamine (0.3 mg/mL) were purchased from Wako Pure Chemicals (Osaka, Japan).

### HSM and HDI

HSM was prepared from amino acid powders following the formulation of Leibovits-15 medium (Life Technologies) [18]. This HSM lacked arginine, tyrosine, glucose, and sodium pyruvate, but was supplemented with galactose (900 mg/L), ornithine (1 mM), glycerol (5 mM), and proline (260 mM) (all from Wako Pure Chemicals). Proline (30 mg/L) was added for DNA synthesis to occur [21]. Aspartic acid was not included since it is one of the products of ornithine and an arginine substrate. KSR (Life Technologies) was added at a final concentration of 10%, and used instead of fetal calf serum to establish defined xeno-free conditions.

HDI was prepared by mixing oncostatin M (20 ng/mL), FPH1, M50054 (100 μg/mL), non-essential amino acids (glycine, 7.5 mg/L; L-alanine, 8.9 mg/L; L-asparagine, 13.2 mg/L; L-aspartic acid, 13.3 mg/L; L-glutamic acid, 14.7 mg/L; L-proline, 11.5 mg/L; and L-serine, 10.5 mg/L), sodium pyruvate (1 mM), nicotinamide (1.2 mg/mL), proline (30 ng/mL), and glutamine (0.3 mg/mL). Proline and nicotinamide are necessary for primary hepatocytes to proliferate [21, 22].

### Media without glucose and arginine, supplemented with galactose and ornithine

HDI was made from powder as previously described [20]. Medium without glucose and arginine, but supplemented with galactose and ornithine, was made from a powder based on the formulae of William’s E (WE) medium and Dulbecco's Modified Eagle's Medium/Nutrient F-12 Ham (DF12), giving rise to modified WE (mWE) and DF12 (mDF12), respectively. HDI, mWE, and mDF12 were further supplemented with proline, nicotinamide, oncostatin M, FPH1, non-essential amino acids, sodium pyruvate, M50054, and 10% KSR.

### Counting cell numbers

201B7 cells were cultured on 6-well plates (Asahi Techno Glass) coated with Matrigel^TM^. When the cells reached confluency, the media was replaced with HDI. After 2 days of culture in HDI, the media was again replaced with L15, WE, or DF12. After 5 days, the cells were harvested with Accutase, and stained with Trypan Blue (Sigma-Aldrich, St. Louis, MO, USA). Unstained cells were counted by microscopy (CKX41N-31PHP).

### Real-time quantitative polymerase chain reaction (qPCR)

Five micrograms of total RNA, isolated with Isogen (Nippon Gene, Tokyo, Japan) were used for the synthesis of first-strand cDNA with SuperScript III reverse transcriptase and oligo (dT) primers (Life Technologies), following the manufacturer’s instructions. Total RNA from human fetal liver was purchased from Clontech (Mountain View, CA, USA). Real-time qPCR was performed with Fast SYBR Green Master Mix (Life Technologies), and the results analyzed using the Mini Opticon system (Bio-Rad, Hercules, CA, USA). Real-time qPCR was performed for 40 cycles, with a 5 s of denaturation and annealing, each. The primer sequences are presented in Table 2. *RPL19* was used as endogenous control to monitor the amount of mRNA since it is a constitutively expressed house-keeping gene [24]. The gene expression levels were analyzed automatically by the Mini Opticon system based on the delta-delta cycle threshold (ddCt) method [25]. The relative expression was calculated as the expression level of a specific gene divided by that of *RPL19*.

**Table 2:** Sequences of primers used in this study.

Primer name	Sequence	Description	Product size (bp)	Annealing temperature (°C)	Cycles	GenBank accession no.
OMC317	5′-ACACAAAAAGCCCACTCCAG-3′	AFP, forward	147	60	40	NM_001134
OMC318	5′-GGTGCATACAGGAAGGGATG-3′	AFP, reverse	147	60	40	
OMC329	5′-GCTCGTGAAACACAAGCCCAAG-3′	ALB, forward	114	60	40	NM_000477
OMC330	5′-GCAAAGCAGGTCTCCTTATCGTC-3′	ALB, reverse	114	60	40	
OMC579	5′-AACAGAGCCAGTCACAGCACCAAG-3′	G6P, forward	139	60	40	NM_000151
OMC580	5′-CCTCAGGAAATCCATTGATACGG-3′	G6P, reverse	139	60	40	
OMC527	5′-TGAGAAATCTGAGGCGGGAAGC-3′	CYP3A4, forward	111	60	40	J04449
OMC528	5′-CGATGTTCACTCCAAATGATGTGC-3	CYP3A4, reverse	111	60	40	
OMC537	5′-GTTACTTCATCCAGCCCACTGTG-3′	ALDH2, forward	121	60	40	AY621070
OMC538	5′-CCAACAACCTCCTCTATGGTCTTG-3′	ALDH2, reverse	121	60	40	
OMC321	5′-CGAATGCCAGAGAAGGTCAC-3′	RPL19, forward	157	60	40	BC095445
OMC322	5′-CCATGAGAATCCGCTTGTTT-3′	RPL19, reverse	157	60	40	

AFP, α-feto protein; ALB, albumin; G6P, glucose-6-phosphatase; CYP3A4, cytochrome P-450 family 3 subfamily A polypeptide 4; ALDH2, aldehyde dehydrogenase 2; RPL19, ribosomal protein L (*RPL*) 19.

### Immunostaining

201B7 cells were cultured on 8-well chamber slides (Matsunami, Kishiwada, Japan) coated with Matrigel^TM^. After culture, the cells were fixed with 4% paraformaldehyde (Sigma-Aldrich) at 4°C for 10 min. The fixed cells were incubated with 0.1% hydrogen peroxide in 100% methanol (both from Wako Pure Chemicals) at 4°C for 30 min to inactivate the endogenous peroxide. After rinsing with phosphate-buffered saline (PBS), the specimens were incubated with wash buffer (2% fetal bovine serum in PBS) at 4°C for 30 min. Either monoclonal anti-human AFP antibody raised in mouse (Takara, Ohtsu, Japan) or polyclonal anti-human albumin (ALB) antibody raised in rabbit (Sigma-Aldrich) was applied to the specimens in wash buffer at 1:1000 dilution. The samples were then incubated at 4°C overnight after which, they were washed with PBS. They were then incubated with either alkaline phosphatase-linked anti-mouse antibody raised in goat (Promega, Madison, WI, USA) or alkaline phosphatase-linked anti-rabbit antibody raised in goat (Promega) in wash buffer at 1:1000 dilution, at 4°C for 2 h. After washing with PBS, the conjugates were visualized using Vector Red Substrate (Vector Laboratories, Burlingame, CA, USA) according to the manufacturer’s instructions. The specimens were then observed under a microscope (AX80) (Olympus, Tokyo, Japan).

### Indocyanine Green uptake assay

Indocyanine Green (ICG; 25 mg; Dai-ichi Pharm, Tokyo, Japan) was dissolved in 5 mL of water. Of this, 1 mL was added to 4 mL of culture media. The final concentration of ICG was 1 mg/mL. The cells were incubated in the media with ICG at 37°C for 15 min. The cells were rinsed with PBS, and observed by microscopy (CKX41N-31PHP).

### Statistical analyses

One-way analysis of variance (ANOVA) was applied for statistical analysis with JMP 10.0.2 (SAS Institute, Cary, NC). P values <0.05 were considered statistically significant.

## Results

**Fig 1:**
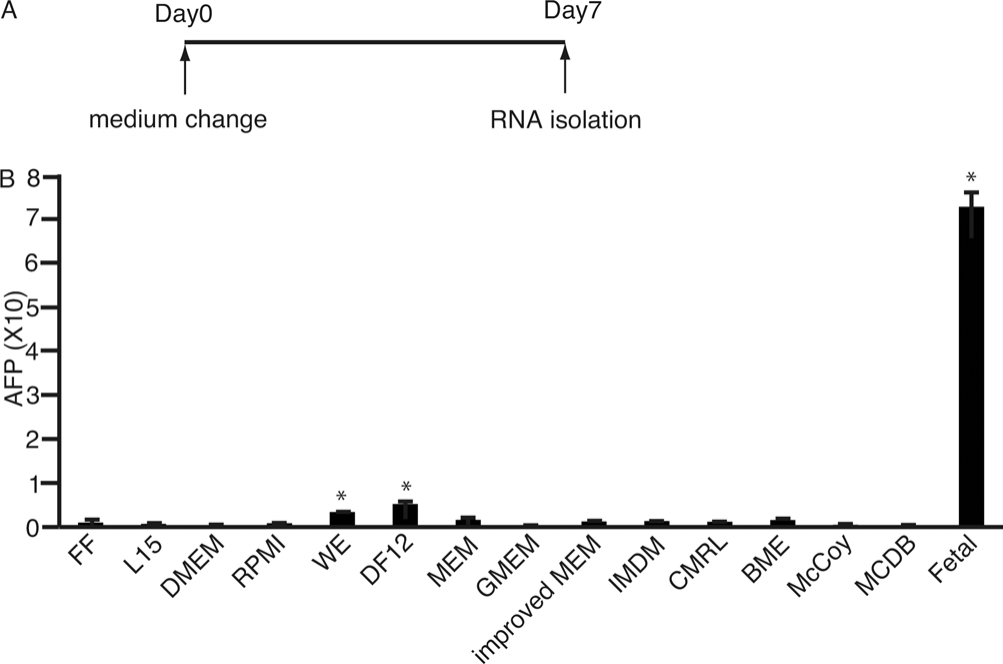
Culture of 201B7 cells in conventional media. 201B7 cells were cultured in different conventional media for 7 days, and then subjected to real-time quantitative polymerase chain reaction to analyze the expression of α-feto protein (AFP). The expression levels of AFP were significantly higher in the cells in WE, DF12 and fetal liver than those in ReproFF (P < 0.05, one-way analysis of variance, n = 3). FF, ReproFF; L15, Leibovitz's-15; DMEM, Dulbecco's Modified Eagle's Medium; RPMI, Roswell Park Memorial Institute 1640; WE, William’s E; DF12, Dulbecco's Modified Eagle's Medium/Nutrient F-12 Ham; MEM, Minimum Essential Medium; GMEM, Glasgow’s Minimum Essential Medium; IMDM, Iscove’s Modified Dulbecco’s Medium; CMRL, Connaught Medical Research Laboratories 1066; BME, Basal Medium Eagle; McCoy, McCoy’s 5A; MCDB, MCDB 131; fetal, fetal liver. Data are presented as the mean ± standard deviation (error bars). *, The expression levels of AFP were higher in the cells in WE and DF12 and fetal liver than those in ReproFF with P <0.05 (one-way analysis of variance).

201B7 cells did not survive in HDI beyond 7 days [20]. It was expected that 201B7 cells survive in conventional media after culture in HDI. If the expression levels of AFP increased in conventional media, the media would be more suitable for the differentiation of 201B7 cells to hepatocyte lineage. To investigate the media for increased expression of AFP, 201B7 iPS cells were cultured in conventional media for 7 days (Fig 1A). HDI was not analyzed because enough RNA was not obtained from 201B7 cells cultured in HDI for 7 days. RNA was isolated and subjected to qPCR. The expression levels of *AFP* were higher in the cells cultured in WE as well as in DF12 (Fig 1B). These results suggest that both WE and DF12 either initiated or promoted differentiation of the 201B7 cells into hepatocytes. Based on these initial results, WE and DF12 were subjected to further analyses. L15 was also tested since the medium was found to enhance the survival of hepatocytes [26].

**Fig 2:**
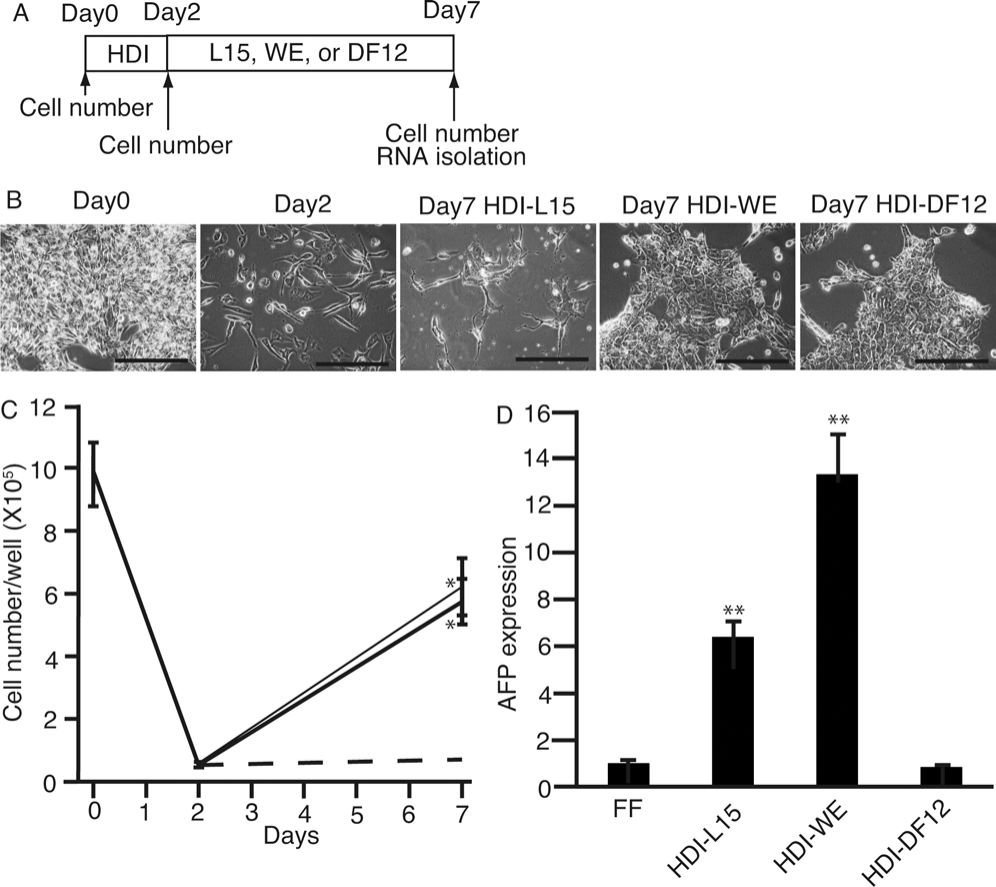
Culture of 201B7 cells in conventional media following that in HDI. **(A)** 201B7 cells were cultured in HDI for 2 days, followed by L15, WE, or DF12 for 5 days. **(B)** Cells were imaged on days 0, 2, and 7. **(C)** Numbers of the cells were counted on days 0, 2, and 7. Cells survived more in HDI-WE (thin-solid line) and HDI-DF12 (thick-solid line) than HDI-L15 (broken line). The numbers of the cells were significantly higher in the cells in HDI-WE and HDI-DF12 than those in HDI-L15 (*: P < 0.05, one-factor analysis of variance, n = 3). **(D)** RNA was isolated, and subjected to real-time quantitative polymerase chain reaction to analyze the expression of α-feto protein (AFP). The expression levels of AFP were significantly higher in the cells in HDI-WE and HDI-DF12 than those in ReproFF (**: P < 0.05, one-factor analysis of variance, n = 3). HDI: hepatocyte differentiation inducer, L15: Leibovitz’s-15, WE: William’s E, DF12: Dulbecco's Modified Eagle's Medium/Nutrient F-12 Ham, FF, ReproFF; HDI-L15, HDI followed by L15; HDI-WE, HDI followed by WE; HDI-DF12, HDI followed by DF12. Data are presented as the mean ± standard deviation (error bars). Original magnification, ×200; scale bar, 100 μm. *, the numbers of the cells were higher in HDI-WE and HDI-DF12 than HDI-L15 with P <0.05 (one-factor analysis of variance), n = 3; **, the expression levels of *AFP* was higher in the cells in HDI-WE and HDI-DF12 than those in ReproFF with P <0.05 (one-factor analysis of variance), n = 3.

Next, to address the possibility that L15, WE, and DF12 enhance survival, 201B7 cells were cultured in HDI for 2 days, followed by another 7 days of culture in the three media (Fig 2A). After 2 days of culture in HDI, the medium was changed to L15, WE, or DF12, and cultured for another 7 days. The morphologies were similar for the cells cultured in L15, WE, and DF12 in the presence of HDI (Fig 2B), but not that of typical hepatocytes—binucleated polygonal. The number of cells decreased to 5.4 ± 0.6% (mean ± standard deviation) as compared to that of day 0. The number of cells recovered to 63.4 ± 9.3% in WE and 58.5 ± 7.4% as compared to that of day 0. After 7 days culture, more cells survived in WE and DF12 when compared with that in L15 (Fig 2C). Surviving cells were subjected to qPCR to analyze the expression of *AFP* (Fig 2D). 201B7 cells cultured in WE exhibited the highest expression levels of *AFP*. These results suggest that HDI, followed by WE, either initiates or promotes differentiation of 201B7 cells to hepatocytes.

**Fig 3:**
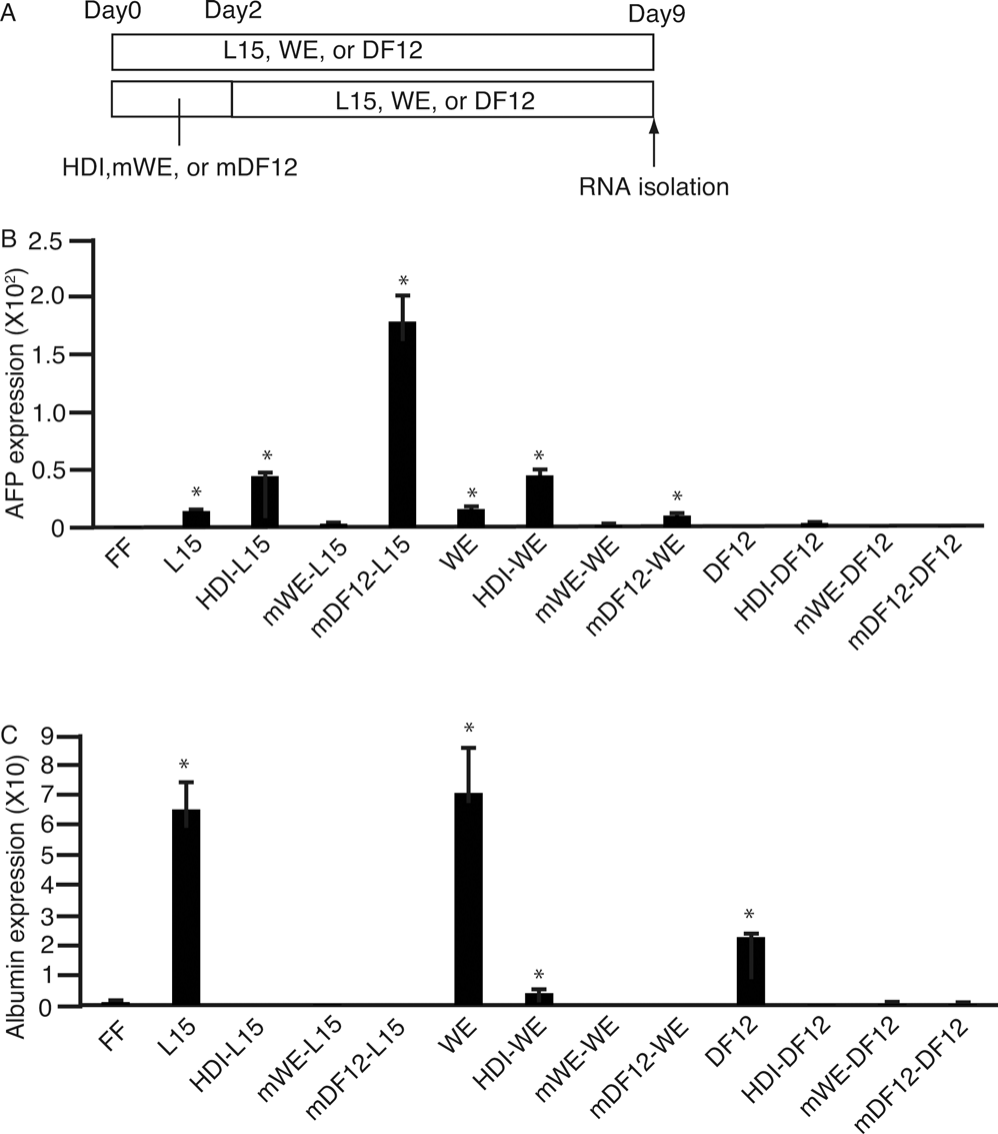
Culture of 201B7 cells in media without glucose and arginine, but supplemented with galactose and ornithine, followed by that in conventional media. **(A)** 201B7 cells were cultured in media without glucose and arginine, but supplemented with galactose and ornithine HDI, mWE, or mDF12), for 2 days, followed by 7 days in L15, WE, or DF12. Cells cultured for 9 days in L15, WE, or DF12 only were used to compare with those in HDI, mWE, and mDF12. **(B and C)** RNA was isolated and subjected to real-time quantitative polymerase chain reaction to analyze the expression of α-feto protein (*AFP*) **(B)** and albumin (*ALB*) **(C)**. HDI, hepatocyte differentiation inducer; WE, William’s E; DF12, Dulbecco's Modified Eagle's Medium/Nutrient F-12 Ham; mWE, modified William’s E; mDF12, modified Dulbecco's Modified Eagle's Medium/Nutrient F-12 Ham; FF, ReproFF; HDI-L15, HDI followed by L15; mWE-L15, mWE followed by L15; mDF12-L15, mDF12 followed by L15; HDI-WE, HDI followed by WE; mWE-WE, mWE followed by WE; mDF12-WE, mDF12 followed by WE; HDI-DF12, HDI followed by DF12; mWE-DF12, mWE followed by DF12; mDF12-DF12, mDF12 followed by DF12. Data are presented as the mean ± standard deviation (error bars). *, P < 0.05 compared with FF (one-way analysis of variance); n = 3.

We could not rule out the possibility that culture in either WE or DF12, which are deprived of glucose and arginine but supplemented with galactose and ornithine, initiated the differentiation of 201B7 cells to hepatocytes. To address this, we modified these two media to mWE and mDF12. Similar to HDI, mWE and mDF12 were supplemented with M50054 and the aforementioned reagents. 201B7 cells were cultured in HDI, mWE, or mDF12 for 2 days, followed by 7 days in L15, WE, or DF12 (Fig 3A). After 7 days, RNA was isolated and subjected to qPCR for *AFP* (Fig 3B) and *ALB* (Fig 3C). mDF12-L15 showed the highest expression for *AFP*, but none for *ALB*.

**Fig 4:**
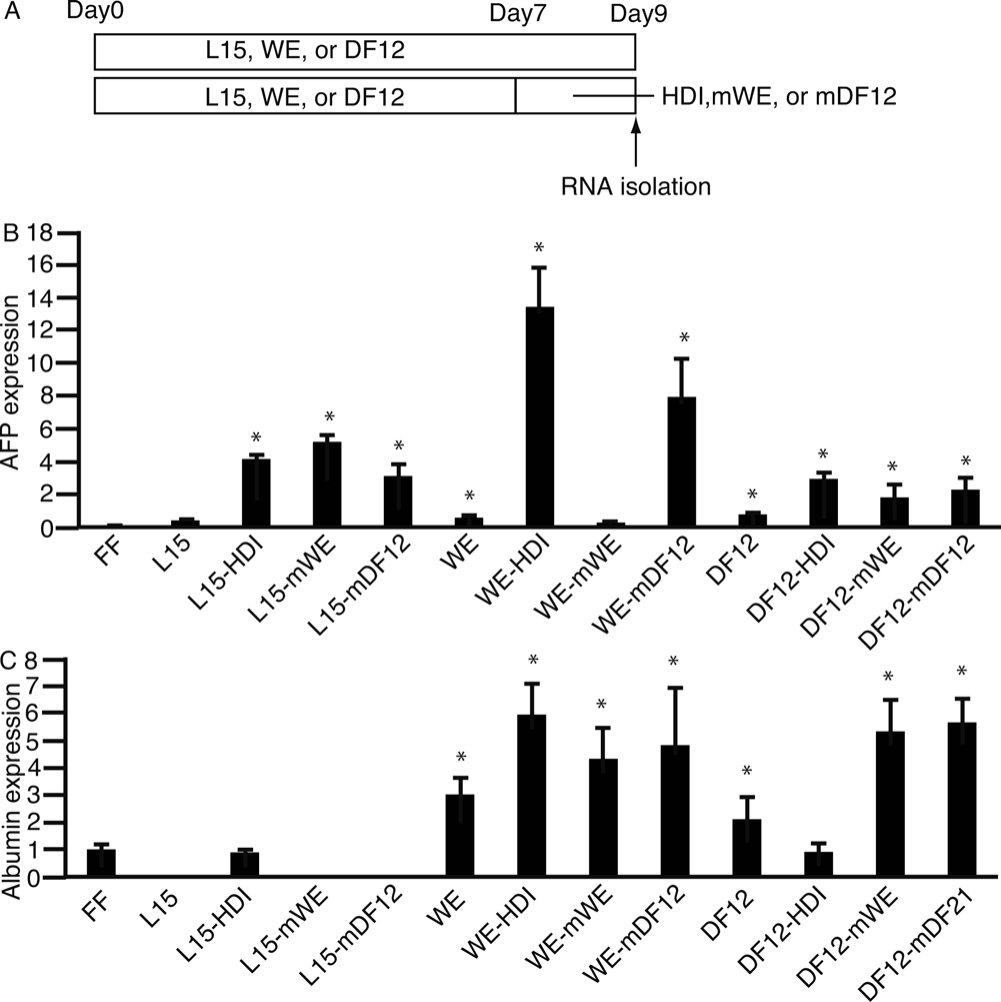
Culture of 201B7 cells in conventional media followed by that in media without glucose and arginine, but supplemented with galactose and ornithine. **(A)** 201B7 cells were cultured in L15, WE, or DF12 for 7 days, followed by 2 days in media without glucose and arginine, but supplemented with galactose and ornithine (HDI, mWE, or mDF12). Cells cultured for 9 days in L15, WE, or DF12 only were used to compare with those in HDI, mWE, and mDF12. **(B and C)** RNA was isolated, and subjected to real-time quantitative polymerase chain reaction to analyze the expression of α-feto protein (*AFP*) **(B)** and albumin (*ALB*) **(C)**. HDI, hepatocyte differentiation inducer; WE, William’s E; DF12, Dulbecco’s Modified Eagle’s Medium/Nutrient F-12 Ham; mWE, modified William’s E; mDF12, modified Dulbecco's Modified Eagle's Medium/Nutrient F-12 Ham; FF, ReproFF; L15-HDI, L15 followed by L15; L15-mDF12, L15 followed by mDF12; WE-HDI, WE followed by HDI; WE-mWE, WE followed by mWE; WE-mDF12, WE followed by mDF12; DF12-HDI, DF12 followed by HDI; DF12-mWE, DF12 followed by mWE; DF12-mDF12, DF12 followed by mDF12. Data are presented as the mean ± standard deviation (error bars). *, P <0.05 compared with FF; n = 3.

Next, we investigated the culture of 201B7 cells in L15, WE, or DF12 for 7 days, followed by 2 days in HDI, mWE, or mDF12 (Fig 4A). These cells were compared with those cultured for 9 days in L15, WE, or DF12. The expression of *AFP* (Fig 4B) and *ALB* (Fig 4C) were analyzed by qPCR, which were the highest in 201B7 cells cultured in WE in presence of HDI. The expression levels of *ALB* in L15, WE, and DF12 in Fig 3 were higher as compared with Fig 4C.

**Fig 5:**
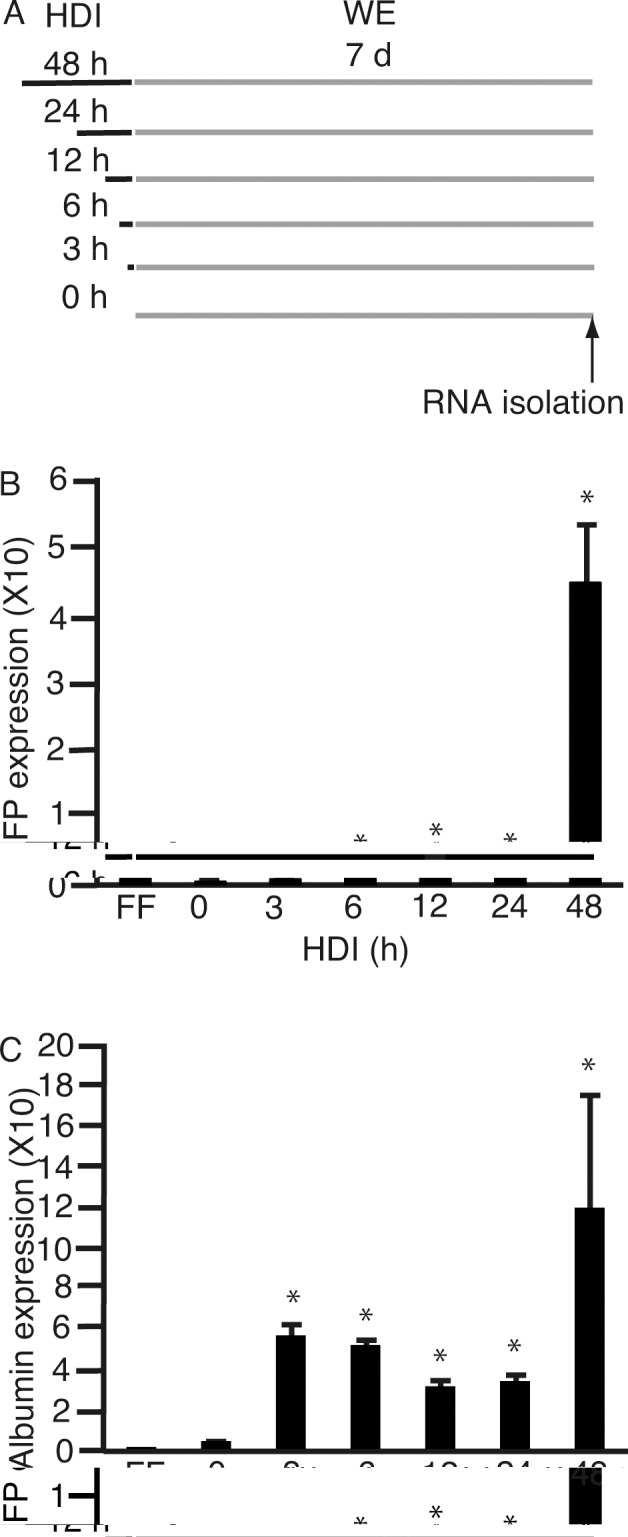
Time-course culture of 201B7 cells in hepatocyte differentiation inducer (HDI). **(A)** 201B7 cells were cultured in HDI (black bar) for 0, 3, 6, 12, 24, and 48 h, followed by another 7 days of culture in William’s E (WE) medium (gray bar). **(B and C)** RNA was isolated, and subjected to real-time quantitative polymerase chain reaction to analyze the expression of α-feto protein (*AFP*) **(B)** and albumin (*ALB*) **(C)**. Data are presented as the mean ± standard deviation (error bars). *, P <0.05 compared with ReproFF (FF); n = 3.

The next issue we addressed was the optimal incubation period for culture in HDI. 201B7 cells were cultured in HDI for 0, 3, 6, 12, 24, and 48 h, followed by 7 days in WE (Fig 5A). RNA was isolated and subjected to qPCR for *AFP* (Fig 5B) and *ALB* (Fig 5B). The highest expression levels of both *AFP* and *ALB* were observed at 48 h, followed by 12 h and 3 h, respectively. Time-course analyses of *AFP* expression was necessary in this case because AFP is abundantly expressed in human fetal livers [27]. The expression levels of *AFP* peaked at 12 h and 48 h. We speculated that incubation in HDI for 12 h played a role in the initiation of differentiation to hepatocyte-lineage. We, thus, compared the results of incubation for 12 h and 48 h in HDI.

**Fig 6:**
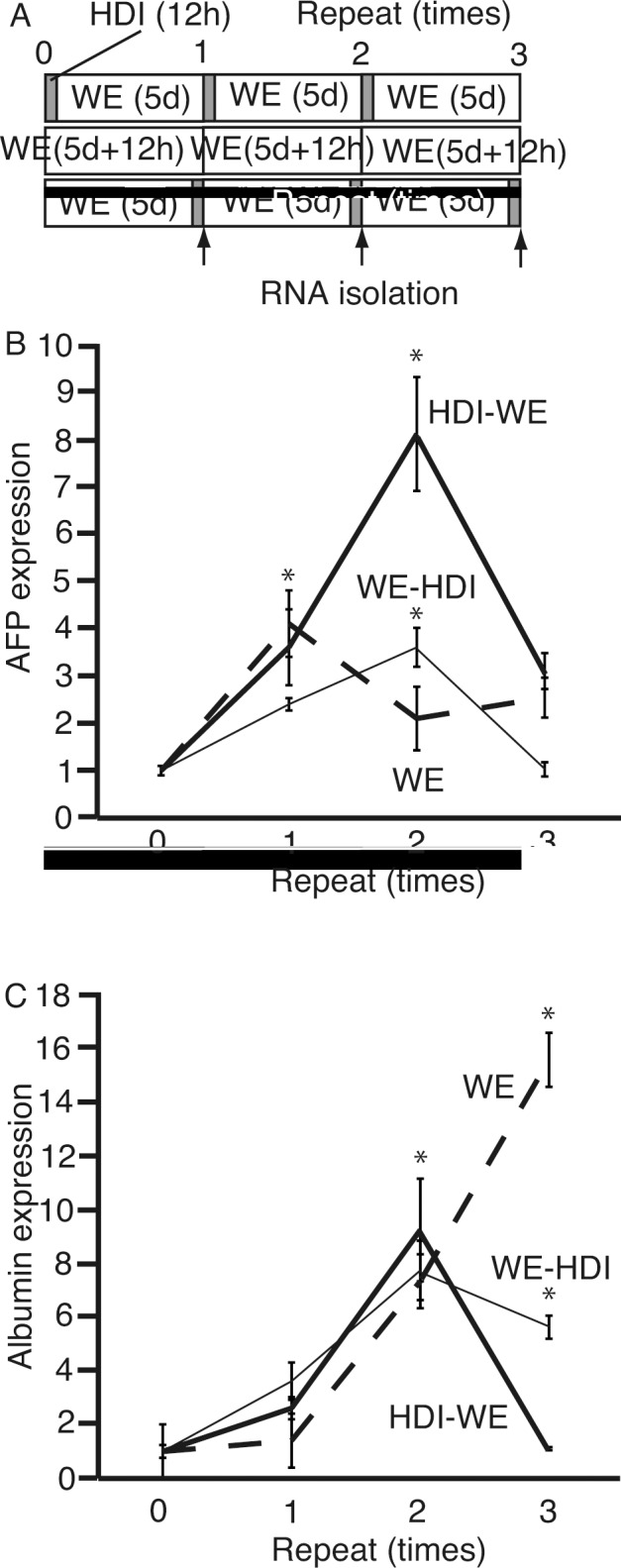
Repeated cycles of 12-h culture of 201B7 cells in hepatocyte differentiation inducer (HDI) either before or after culture in William’s E (WE) medium. **(A)** 201B7 cells were cultured in three conditions: HDI for 12 h followed by WE for 5 days; WE only for 12 h + 5 days; and WE for 5 days followed by HDI for 12 h. **(B and C)** After one, two, or three cycles of these culture conditions, RNA was isolated and subjected to real-time quantitative polymerase chain reaction to analyze the expression of α-feto protein (*AFP*) **(B)** and albumin (*ALB*) **(C)**. The thick solid line indicates HDI followed by WE only, broken line indicates WE only, and thin solid line indicates WE followed by HDI. Data are presented as the mean ± standard deviation (error bars). *, P <0.05 compared with 0 time of repeat; n = 3.

We next hypothesized that repeated culture of cells in HDI followed by that in WE might enhance the differentiation of 201B7 cells into hepatocytes. Initially, HDI was thought to initiate the differentiation of iPS cells to hepatocytes [20]. To confirm that culture in WE followed by HDI is more efficient in initiating differentiation of iPS cells to hepatocyte-lineage, we analyzed culture of the cells in HDI followed by WE. 201B7 cells were cultured in cycles of any of the following three conditions: HDI for 12 h followed by WE for 5 days; WE only for 12 h + 5 days; and WE for 5 days followed by HDI for 12 h (Fig 6A). RNA was isolated after 0, 1, 2, and 3 cycles, followed by qPCR for *AFP* (Fig 6B) and *ALB* (Fig 6C). The expression of *AFP* was highest in cells cultured for two cycles of HDI followed by WE, while that of *ALB* was highest in cells cultured for three cycles of WE alone. Additionally, the peak expression levels of *AFP* and *ALB* were not same.

**Fig 7:**
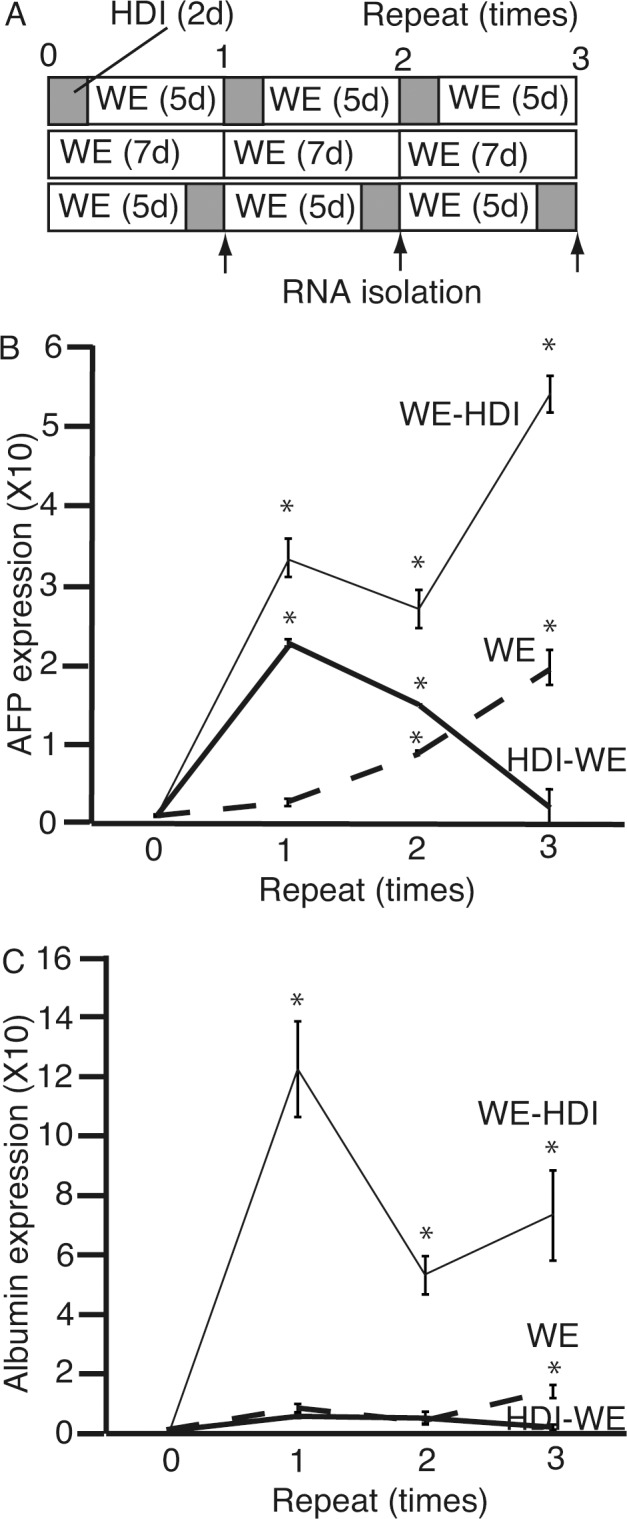
Repeated cycles of 2d culture of 201B7 cells in hepatocyte differentiation inducer (HDI) either before or after culture in William’s E (WE) medium. **(A)** 201B7 cells were cultured in three conditions; HDI for 2 days followed by WE for 5 days, WE only for 7 days, and WE for 5 days followed by HDI for 2 days. **(B and C)** After one, two, or three cycles of these culture conditions, RNA was isolated, and subjected to quantitative polymerase chain reaction to analyze the expression of α-feto protein (*AFP*) **(B)** and albumin (*ALB*) **(C)**. The thick solid line indicates HDI followed by WE, thick broken line indicates WE only, and thin solid line indicates WE followed by HDI. Data are presented as the mean ± standard deviation (error bars). *, P <0.05 compared with 0 time of repeat; n = 3.

Based on the hypothesis that culture in HDI followed by WE enhances the differentiation to hepatocytes, we cultured 201B7 cells in cycles of three different conditions: HDI for 2 days followed by WE for 5 days; WE only for 7 days; and WE for 5 days followed by HDI for 2 days (Fig 7A). Initially, HDI was thought to initiate the differentiation of iPS cells to hepatocytes. To confirm that culture in WE followed by HDI is more efficient in initiating the differentiation of iPS cells to hepatocyte-lineage, we analyzed culture of the cells in HDI followed by WE. RNA was isolated after 0, 1, 2, and 3 cycles and subjected to qPCR for *AFP* (Fig 7B) and *ALB* (Fig 7C). The expression of *AFP* peaked after three cycles, while that of *ALB* peaked after one. Moreover, the relative expression levels of *AFP* and *ALB* were greater than those shown in Fig 6B and 6C, respectively. After three cycles of culture in WE for 5 days followed by 2 days in HDI, the 201B7 cells expressed *AFP* and *ALB* 54 ± 2.3 (average ± standard deviation) and 73 ± 15.1 times higher, respectively, than those cultured in ReproFF (feeder-free conditions). These results suggest that 5 days in WE followed by 2 days in HDI either initiates or promotes the differentiation of 201B7 cells to hepatocytes.

**Fig 8:**
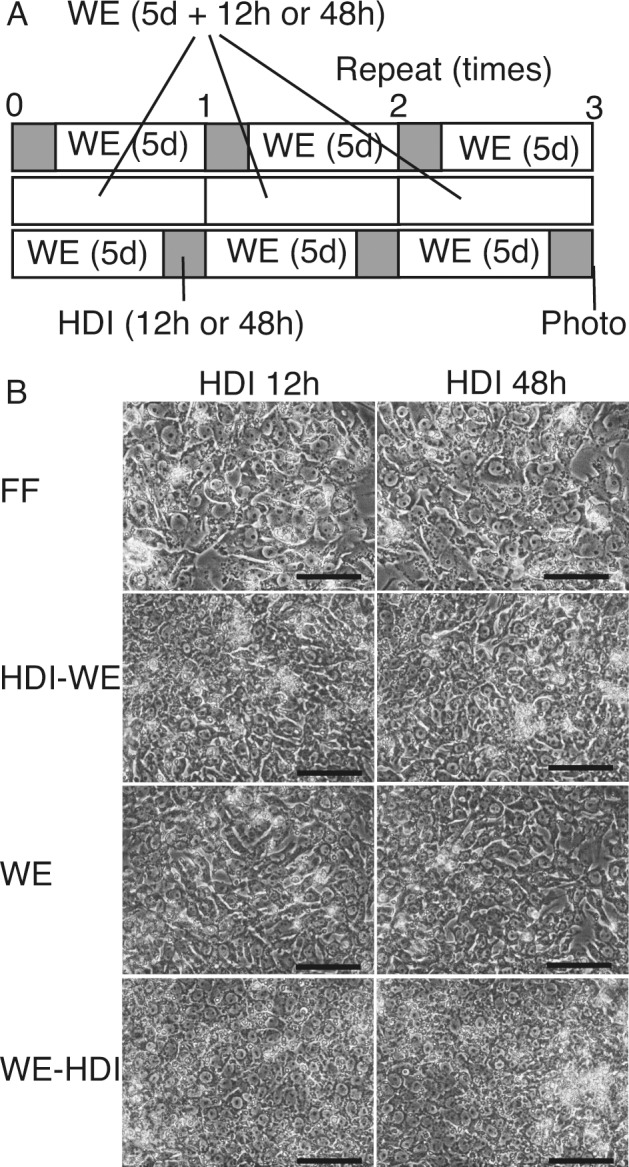
Morphology of 201B7 cells cultured in repeated combinations of hepatocyte differentiation inducer (HDI) and William’s E (WE) medium. **(A)** 201B7 cells were cultured in ReproFF, HDI followed by WE, WE only, or WE followed by HDI. The cells were cultured in HDI for either 12 h or 48 h and then cultured for another 5 days in combination with HID. After three cycles of the culture combinations, the cells were imaged **(B)**. Original magnification, ×400; scale bar, 100 μm.

To observe the morphology of the cells, images were acquired after three cycles of the following three conditions: HDI for either 12 h or 48 h followed by HDI for 5 days; WE only for 5 days + either 12 h or 48 h; and WE for 5 days followed by HDI for 12 h (Fig 8A). None of the conditions yielded typical hepatocytic morphology to the cells—binucleated polygonal (Fig 8B).

**Fig 9:**
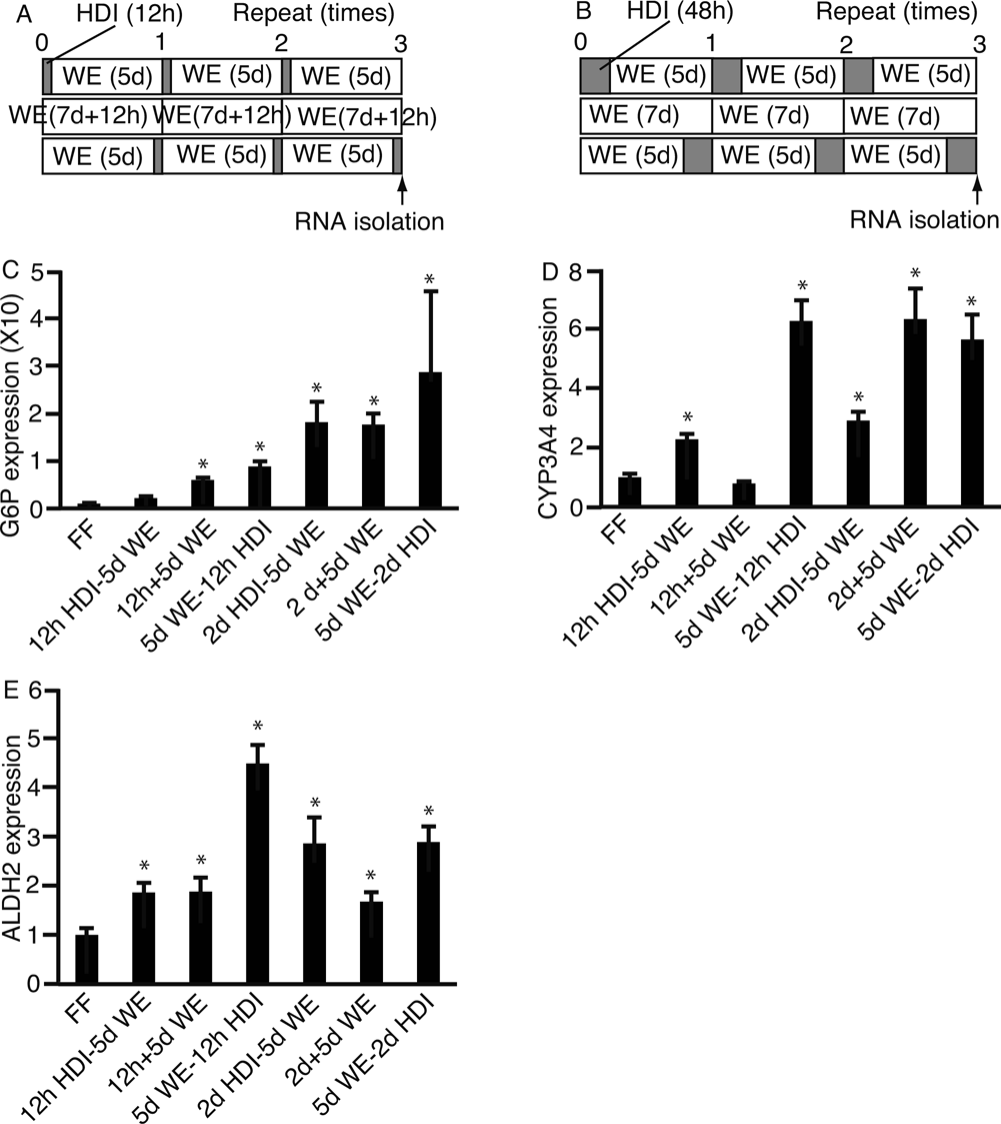
Expression of liver-specific genes in 201B7 cells in presence of HDI. **(A)** 201B7 cells were cultured in three conditions: hepatocyte differentiation inducer (HDI) for 12 h followed by William’s E (WE) medium for 5 days; WE only for 12 h + 5 days; and WE for 5 days followed by HDI for 12 h. **(B)** 201B7 cells were cultured in three conditions: HDI for 2 days followed by WE for 5 days; WE only for 7 days; and WE for 5 days followed by HDI for 2 days. RNA was isolated and subjected to quantitative polymerase chain reaction to analyze the expression of glucose-6-phosphatase (*G6P*) **(C)**, cytochrome P450 family 3 subfamily A polypeptide 4 (*CYP3A4*) (D), and aldehyde dehydrogenase 2 (*ALDH2*) (E). Data are presented as the mean ± standard deviation (error bars). *, P <0.05 compared with ReproFF (FF); n = 3.

Moreover, we next analyzed the expression of liver-specific genes by qPCR after three cycles of the following three conditions; HDI followed by WE; WE only; and WE followed by HDI. 201B7 cells were cultured in HDI for either 12 h (Fig 9A) or 2 days (Fig 9B). Of the genes studied, glucose-6-phosphatase (*G6P*) was the most expressed in 5 days WE-2 days HDI (Fig 9C). Meanwhile, the expression of cytochrome P450 family 3 subfamily A polypeptide 4 (*CYP3A4*) was similar for the 5 days WE-12 h HDI, 2 days + 5 days WE, and 5 days WE-2 days HDI conditions (Fig 9D). Further, aldehyde dehydrogenase 2 (*ALDH2*) was the most expressed in 5 days WE-12 h HDI (Fig 9E), followed by that in 2 days HDI-5 days WE and 5 days WE-2 days HDI. These results suggest that the 5 days WE-2 days HDI condition enhanced liver-specific gene expression the most.

**Fig 10:**
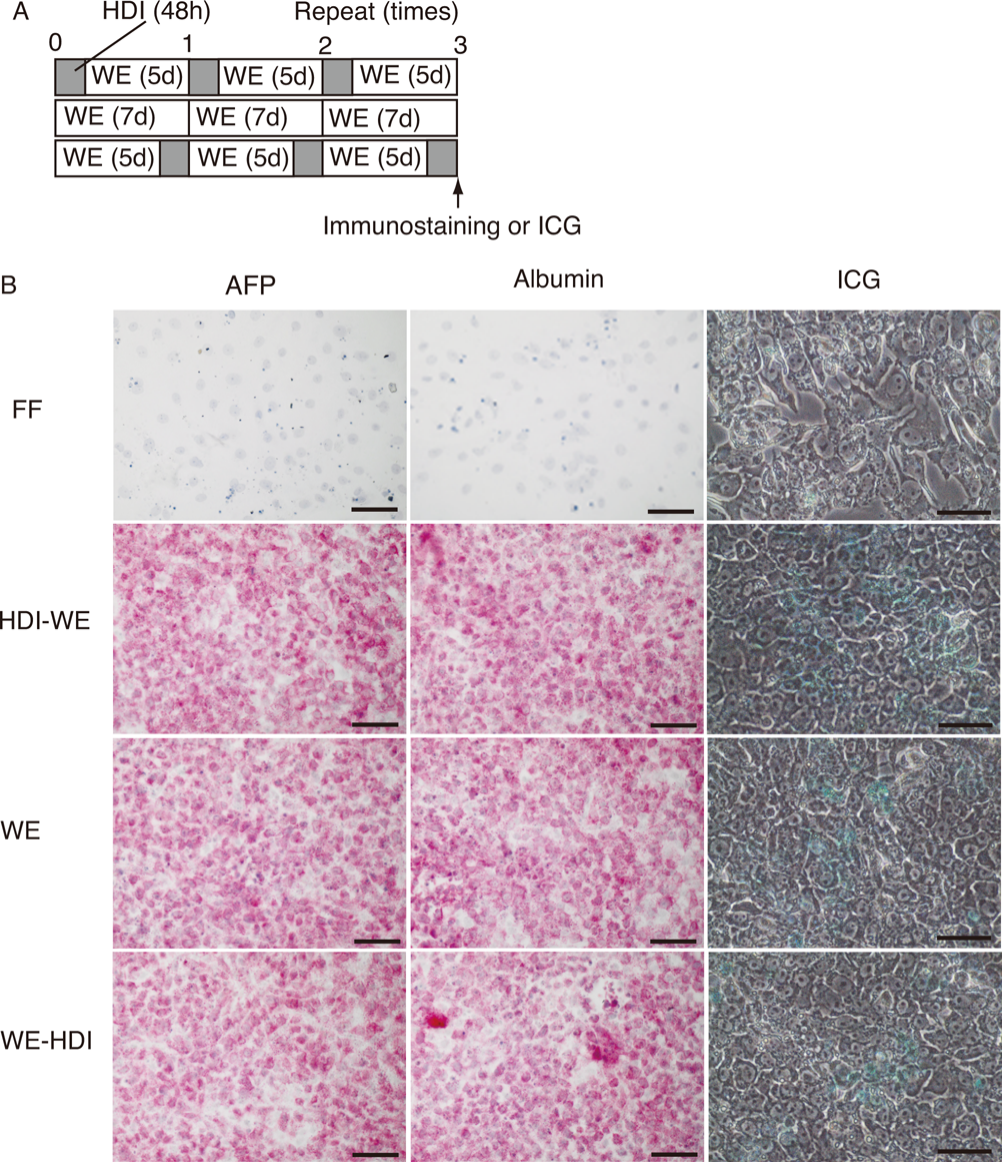
Immunostaining and Indocyanine Green uptake by cells grown in HDI. **(A)** 201B7 cells were cultured in three conditions: hepatocyte differentiation inducer (HDI) for 2 days followed by William’s E (WE) for 5 days (HDI-WE); WE only for 7 days (WE); and WE for 5 days followed by HDI for 2 days (WE-HDI). The cells cultured in ReproFF (FF) were considered undifferentiated controls. **(B)** The cells were immunostained with antibodies against α-feto protein (AFP) and albumin (ALB). The cells were subjected to Indocyanine Green uptake (ICG). Original magnification was ×400 and scale bars represent 50 μm for both immunostaining and ICG uptake.

To analyze the expression of AFP and ALB proteins, we performed immunostaining. ICG is taken up specifically by hepatocytes, therefore, its uptake assay was performed to analyze the hepatocyte-specific function of the cultured cells. Both immunostaining and ICG uptake assay were performed after three cycles of the following three conditions: HDI followed by WE; WE only; and WE followed by HDI (Fig 10A). Given our earlier observation that a 2-day incubation in HDI more efficiently enhanced AFP and ALB expression (Figs 6 and 7), we applied HDI to the cells for 2 days in this experiment as well. Expression of both AFP and ALB were stronger in cells cultured in HDI-WE, WE, and WE-HDI when compared with that in ReproFF (Fig 10B). Similarly, higher ICG uptake was also observed in the cells cultured in HDI, WE, and WE-HDI compared with that in ReproFF (Fig 10B). Overall, these results suggest enhanced expression of AFP and ALB as well as hepatocyte-specific function in cells cultured in HDI-WE, WE, and WE-HDI.

## Discussion

HDI initiates differentiation of iPS cells into hepatocytes [20]. iPS cells express liver-specific genes after 2 days of culture in HDI. A characteristic unique to HDI is that it does not contain either glucose or arginine, and is supplemented with galactose and ornithine. Other conventional media contain both glucose and arginine since they are essential for cultured cells to survive. The deprivation of glucose and arginine, and the supplementation of galactose and ornithine, might promote the differentiation of iPS cells into hepatocyte-like cells [28]. In fact, cells grown under these conditions have been known acquire drug metabolism profiles similar to hepatocytes [28]. These reports indicate that iPS cells can differentiate into the hepatocyte lineage in media without glucose and arginine, but supplemented with galactose and ornithine.

Details of the mechanism underlying differentiation of iPS cells into the hepatocyte-lineage remains largely unknown. Notably, only hepatocytes can convert galactose into glucose and ornithine into arginine through gluconeogenesis and the urea cycle, respectively. One speculation is that iPS cells that undergo differentiation into hepatocytes to acquire gluconeogenesis and the urea cycle are capable of survival. Meanwhile, galactose enhances the survival of hepatocytes as well as their attachment to three dimensional scaffolds [29]. It also enhances the differentiation of human embryonic stem cells into hepatocyte-like cells [30]. These results indicate that galactose is involved in the survival and hepatocyte differentiation of iPS cells. The underlying mechanism(s), however, remains unknown.

However, one major limitation of using HDI is that cells that have initiated differentiation to hepatocytes do not survive beyond 7 days. Another limitation is the lack of ALB expression, suggesting that iPS cells remain immature [20]. To address these limitations in the current study, we investigated a combination of conventional-media culture conditions. Culture of cells in WE followed by HDI yielded the greatest increase in AFP and ALB expression. After three cycles of this media combination, cells survived and expressed both AFP and ALB. This indicates that three cycles of culture in WE followed by HDI is suitable for iPS cells to differentiate into the hepatocyte lineage. Rat primary hepatocytes are reportedly capable of maintaining drug metabolism in WE [31, 32]. Gluconeogenesis and urea synthesis are also preserved in hepatocytes cultured in WE [33, 34]. These studies demonstrate that WE is the most suitable medium for the functional maintenance of cultured hepatocytes, and their results are consistent with those of the present study.

In this study, there were inconsistencies in the qPCR results. The expression of *ALB* in HDI-WE was lower in Fig 3C than that in Fig 5C. The expression levels of *AFP* in HDI-L15 and HDI-WE also differed between Figs 2C and 3B. Further, the expression levels of *ALB* in L15, WE, and DF12 in Fig 3 were higher as compared with Fig 4. The reason behind these inconsistencies is not clear. Results of qPCR are sometimes inconsistent even when the same samples are analyzed using the same instruments [35]. Small number of copy of mRNA might also affect consistency of the results [36]. Moreover, constant absolute threshold for ddCt estimation might cause some variability [37].

In the present study, growth factors were not added in the conventional media. Most of the protocols use growth factors to promote the differentiation of iPS cells to hepatocytes [5, 6]. Absence of growth factors might be one of the reasons why the cells did not have the typical morphology of hepatocytes after culture in WE followed by HDI. It would be expected that growth factor promotes differentiation of iPS cells to hepatocytes with typical morphological features. Our next step would be to search for growth factors that promoted hepatocyte differentiation of iPS cells in WE followed by HDI.

In conclusion, 201B7 cells survived the combination of 2-day culture in HDI following 5 days in WE. After three cycles of culture in WE for 5 days followed by 2 days in HDI, hepatocyte differentiation was enhanced as reflected by increased expression of both AFP and ALB.
